# NADPH Oxidase-Induced NALP3 Inflammasome Activation Is Driven by Thioredoxin-Interacting Protein Which Contributes to Podocyte Injury in Hyperglycemia

**DOI:** 10.1155/2015/504761

**Published:** 2015-03-05

**Authors:** Pan Gao, Fang-Fang He, Hui Tang, Chun-Tao Lei, Shan Chen, Xian-Fang Meng, Hua Su, Chun Zhang

**Affiliations:** ^1^Department of Nephrology, Union Hospital, Tongji Medical College, Huazhong University of Science and Technology, Wuhan 430022, China; ^2^Department of Neurobiology, Tongji Medical College, Huazhong University of Science and Technology, Wuhan 430030, China

## Abstract

Diabetic nephropathy (DN) is one of the major causes of end-stage renal disease, and previously we demonstrated that NALP3 inflammasome was involved in the pathogenesis of DN. Here we investigated the mechanisms of NALP3 inflammasome activation in podocyte injury during DN. We found that, besides the activation of NALP3 inflammasome and upregulated thioredoxin-interacting protein (TXNIP), the glomerular expression of gp91^*phox*^, a subunit of NADPH oxidase, was enhanced in DN mice simultaneously. Inhibiting NADPH oxidase abrogated NALP3 inflammasome activation, and IL-1*β* production and eventually protected podocytes from high glucose- (HG-) induced injury. TXNIP, an inhibitor of thioredoxin, acts as a suppressor for antioxidant defense system. Our observation indicated that in HG-exposed podocytes genetic deletion of TXNIP by shRNA reversed gp91^*phox*^ overexpression and alleviated the injury of podocyte. Collectively, our findings proposed that HG-induced NADPH oxidase activation was driven by TXNIP which subsequently triggered NALP3 inflammasome activation in podocytes and ultimately led to podocyte injury, and blocking TXNIP/NADPH oxidase signaling may be a promising treatment for DN.

## 1. Introduction

Growing evidences indicated that the elevated reactive oxygen species (ROS) plays an important role in the development and progression of DN* in vivo* and* in vitro *[1–3]. Overproduction of ROS leads to podocyte apoptosis and loss [4, 5], and in the presence of high glucose (HG), ROS is accumulated and results in podocyte dysfunction and increased excretion of albumin in urine [6]. However, the mechanism mediating ROS accumulation and consequent podocyte injury in DN has not been completely clarified yet.

NADPH oxidase, composed of membrane-bound gp91^*phox*^ and p22^*phox*^, cytosolic subunits such as p47^*phox*^ and p67^*phox*^, and the small GTPase Rac, is the main enzyme that catalyzes ROS production in glomeruli under physiological conditions [7]. In renal resident cells, the central mechanisms of NADPH oxidase activation are gp91^*phox*^ upregulation and p47^*phox*^ membranous translocation which result in superoxide anion (O_2_^•−^) aggregation and ultimately lead to podocyte injury and glomerular sclerosis [8, 9].

Recently, NALP3 inflammasome activation is well documented in various renal diseases [10–12]. NALP3 recruits the adaptor molecule-apoptosis associated speck-like protein (ASC) by pyrin domain, and then ASC hydrolyzes procaspase-1. Finally, active caspase-1 cleaves pro-IL-1*β* into its mature form [13, 14]. IL-1*β* is an cardinal proinflammatory cytokine, which governs the outcome of renal disease, and moreover, inhibition of IL-1*β* can ameliorate type 2 diabetes [15]. ROS is the main mediator for NALP3 inflammasome activation [16], and almost all agonists activate NALP3 inflammasome via inducing ROS production [17, 18]. It is reported that under hyperhomocysteinemia NADPH oxidase can activate NALP3 inflammasome in podocytes, which resulted in the recruitment of immune cells and ultimately rendered glomerular impairment [19]. And blocking ROS by chemical scavengers can effectively suppress NALP3 inflammasome activation [20]. Besides ROS, thioredoxin-interacting protein (TXNIP) is another molecule mediating NALP3 activation. Several investigations suggested that a physical interaction between TXNIP and NLRP3 is initiated in a ROS-sensitive manner which subsequently leads to NALP3 activation [16, 21–23]. Furthermore, TXNIP is known to be implicated in glucose metabolism and pathological processes of diabetes [24, 25]. However in DN the role and association of ROS and TXNIP in NALP3 inflammasome activation remain poorly understood.

In this study we found that NADPH oxidase (gp91^*phox*^) dependent ROS generation was triggered by TXNIP, which may play a crucial role in HG-induced NALP3 inflammasome activation ensuing podocyte and glomerular inflammatory injury.

## 2. Materials and Methods

### 2.1. Human Renal Biopsy Samples

Renal biopsies were performed as part of routine clinical diagnostic observation. The diabetic patients' kidney tissue was sampled by the Department of Pathology, Wuhan Union Hospital. Control sample was obtained from the healthy kidney poles of individuals who underwent tumor nephrectomy without any primary or secondary kidney diseases.

### 2.2. Animals

All experiments were performed according to the guidelines for use and care of laboratory animals of National Institutes of Health (NIH) and approved by the Animal Care and Use Committee (ACUC) of Tongji Medical College. C57BL/6 mice of eight-week-old received a single intraperitoneal injection of streptozotocin (STZ, 150 mg/kg; Enzo Life Sciences, Ann Arbor, MI, USA) to set up DN model. Control mice only received citrate buffer. Blood glucose was monitored weekly by glucometer readings. Mice with serum glucose higher than 16.7 mmol/L were included. 8 weeks after injection, the mice were placed into metabolic cages and the urine was collected before sacrifice. Urinary albumin and creatinine were measured by Auto-Chemistry Analyzer of DIRUI CS-400B (Changchun, Jilin, China). The glomeruli from mice were isolated by the sieving method [9]. Firstly, kidney was flushed with ice-cold Krebs–Henseleit–saline buffer by using an aortal catheter. Next, minced renal cortex was passed through three steel sieves (200, 120, and 80 mm). Lastly, the glomeruli were recovered from the 80 mm sieve, washed, and resuspended in ice-cold Krebs–Henseleit–saline buffer.

### 2.3. Cell Culture

An immortalized human podocyte cell line was cultured as described previously [26]. After the cells became 80% confluence at 33°C, they were transferred to 37°C for 2 weeks to allow differentiation before any experimental manipulations. The cells were starved in 2% FBS media for 12 h before following treatments. The cells were exposed to media containing normal glucose (NG) as a control (5.6 mmol/L D-glucose) or high glucose (HG, 30 mmol/L D-glucose) for indicated times. Moreover, 24.4 mmol/L mannitol plus 5.6 mmol/L D-glucose was employed as an osmotic control.

### 2.4. Genetic Deletion of gp91^*phox*^ and TXNIP

Gp91^*phox*^ shRNA and TXNIP shRNA were purchased from Genechem (Shanghai, China); meanwhile the scrambled shRNA (Genechem, Shanghai, China) was used as a control. Podocytes were transiently transfected with gp91^*phox*^/TXNIP shRNA or scrambled shRNA by lipofectamine 2000 (Invitrogen Corp., Carlsbad, CA, USA) according to the manufacturer's instruction. Two days later, the podocytes were exposed to HG (30 mmol/L) for indicated times.

### 2.5. Western Blot Analysis

Western blotting was performed as we described previously [13]. The primary antibodies were used as follows: rabbit anti-NALP3 (1 : 1000; Protein Tech Group, Chicago, IL), rabbit anti-ASC (1 : 100; Santa Cruz Biotechnology, Santa Cruz, CA, USA), rabbit anti-caspase-1 (1 : 100; Santa Cruz Biotechnology, Santa Cruz, CA, USA), mouse anti-desmin (1 : 1000; Protein Tech Group, Chicago, IL), rabbit anti-synaptopodin (1 : 1000; Protein Tech Group, Chicago, IL), mouse anti-TXNIP (1 : 1000; MBL, International Co, Woburn, MA, USA), goat anti-gp91^*phox*^, and mouse anti-*β*-actin (1 : 10000; Santa Cruz Biotechnology Santa Cruz, CA, USA). The membrane was incubated with primary antibodies overnight at 4°C, followed by incubation with horseradish peroxidase-labeled IgG (1 : 10000) at room temperature for 1 hour. The immunoreactive bands were detected by chemiluminescence methods. Densitometric analysis of the images was performed by using Image J software (NIH, Bethesda, MD, USA).

### 2.6. Real-Time Reverse Transcription Polymerase Chain Reaction

Total RNA from the mouse glomeruli was extracted by TRIzol reagent (Tiangen Biotech, Beijing, China) according to the manufacturer's protocol. Aliquots of total RNA (1 *μ*g) from each sample were reverse-transcribed into complementary DNA (cDNA) according to the instructions (Bio-Rad, Hercules, CA, USA). Then equal amounts of the reverse transcriptional products were subjected to PCR amplification using SYBR Green as the fluorescence indicator on a Bio-Rad iCycler system (Bio-Rad, Hercules, CA, USA). The messenger RNA (mRNA) levels of target genes were normalized to the *β*-actin mRNA levels. The primers used in this study were synthesized by Songon Biotech (Shanghai, China), and the sequences were as follows: TXNIP, forward 5′-TGTGAAGTTACCCGAGTCAAAGC-3′ and reverse 5′-AGCGCAAGTAGTCCAAAGTCT-3′; gp91^*phox*^, forward 5′-CAGGAGTTCCAA-GATGCCTG-3′ and reverse 5′-GATTGGCCTGAGATTCATCC-3′; *β*-actin, forward 5′-GTATGACTCCACTCACGGCAAA-3′ and reverse 5′-GGTCTCGCTCCTGGAA-GATG-3′.

### 2.7. NADPH Activity, Intracellular ROS, Caspase-1 Activity, and IL-1*β* Measurement

For NADPH activity examination, the cells were seeded in 24-well plates, after carefully removing the cell culture medium, 500 *μ*L of dye working solution was added and incubated at 37°C for 20 min. Then dye working solution was removed and 500 *μ*L of 37°C preheated preservation solution was added. A dihydroethidium- (DHE-) based fluorescence spectrometric assay was employed to assess NADPH activity. Before recording ethidium fluorescence NADPH (1 mmol/L) was added immediately using a fluorescence microplate reader (FLX800, Bio-Tek, Winooski, VT, USA). The ethidium fluorescence intensity was used to represent NADPH activity.

Caspase-1 activity was examined by using a commercial kit (Biovision, Mountain View, CA, USA), which was used to represent the activation of NALP3 inflammasome. The data was calculated as the fold changes compared to control group.

IL-1*β* concentration in supernatant was measured by ELISA assay according to the protocol described by the manufacturer (R&D Systems, Minneapolis, MN, USA).

### 2.8. Dual Immunofluorescence Staining

To analyze the abundance and localization of IL-1*β* within the glomeruli of DN patients, we perform dual immunofluorescent staining in frozen tissue. After fixation, the tissue was permeabilized and blocked with 5% donkey serum and then incubated with mouse monoclonal anti-IL-1*β* (1 : 50; Protein Tech Group, Chicago, IL, USA) and goat anti-synaptopodin antibody (1 : 40; Santa Cruz Biotechnology, Santa Cruz, CA, USA) overnight at 4°C. After washing, these slides were incubated with Alexa-488 or Alexa-647-labeled secondary antibodies at room temperature for 1 h. The images were captured by confocal microscopy at identical microscopic settings. Negative control was designed by replacing primary antibody with PBS and no visible fluorescence was detected under this setting (data not shown).

### 2.9. Direct Immunofluorescence Staining of F-Actin

To determine the effect of NALP3 inflammasome activation on cytoskeleton arrangement, podocytes were cultured in 24-well plates the day before transfection. After transfection with TXNIP shRNA, gp91^*phox*^ shRNA, or scrambled shRNA or pretreatment with NADPH oxidase inhibitors, the podocytes were exposed to HG (30 mmol/L) for 48 h. The staining procedure was carried out as we described previously [3].

### 2.10. Statistical Analysis

All of the values were expressed as mean ± SEM. Significant differences among multiple groups were examined by using ANOVA followed by a Student-Newman-Keuls post hoc test. *χ* ^2^ test was used to test the significance of ratio and percentage data. *P* < 0.05 was considered as statistical significance.

## 3. Results

### 3.1. NALP3 Inflammasome Is Activated in the Glomeruli of DN Mice

**Figure 1:**
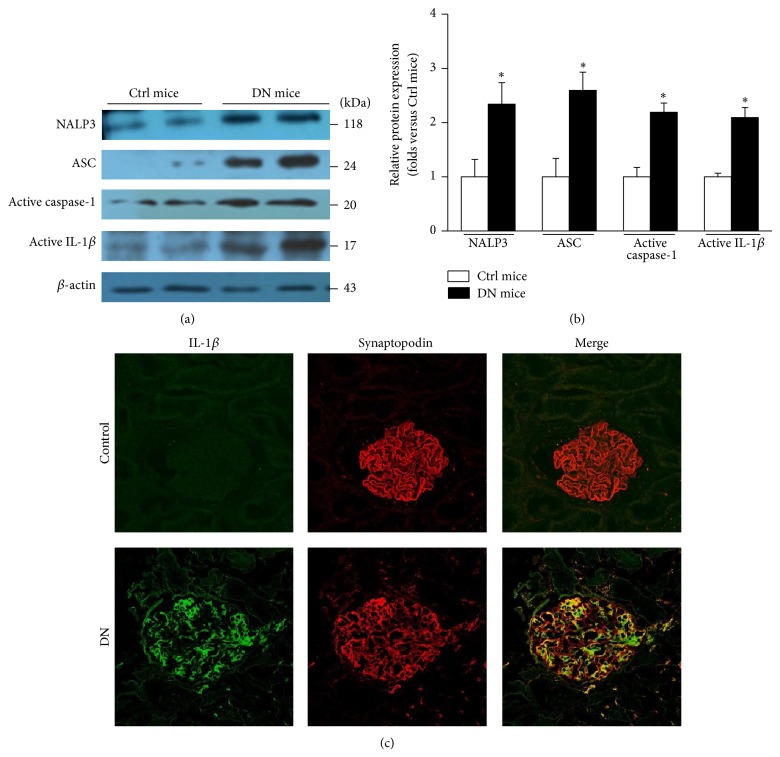
The NALP3 inflammasome is activated in the glomeruli of DN mice. (a) Western blotting gel document showing the expression of NALP3, ASC, active caspase-1, and active IL-1*β* in the glomerular lysate of DN mice. (b) Summarized data presenting the protein expression of NALP3, ASC, active caspase-1, and active IL-1*β* in the glomeruli of DN mice (*n* = 7). Ctrl mice: control mice; DN mice: diabetic nephropathy mice. ^*^ *P* < 0.05 compared with Ctrl mice. (c) Dual immunofluorescence staining of IL-1*β* and synaptopodin in healthy control and DN patients. Original magnification: ×400.

It is well known that the NALP3 inflammasome is involved in several inflammatory renal diseases [10–12], which prompts us to assess the activity of NALP3 inflammasome in DN. Figures 1(a) and 1(b) showed that NALP3, ASC, active caspase-1, and active IL-1*β* proteins were elevated in the glomeruli of DN mice by western blotting. Similarly, in DN patients the abundance of IL-*β* was increased significantly which mainly originated from podocyte as the colocalization analysis shown in Figure 1(c).

### 3.2. Gp91^*phox*^ Is Accumulated in the Glomeruli of DN Mice and HG-Treated Podocyte

**Figure 2:**
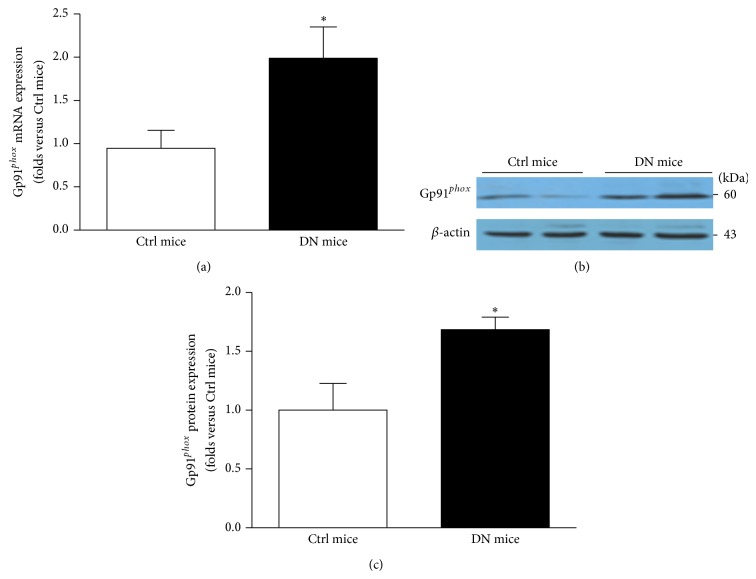
The expression of gp91^*phox*^ is upregulated in the glomeruli of DN mice. (a) Summarized data of gp91^*phox*^ mRNA by RT-PCR in the glomeruli of Ctrl mice and DN mice (*n* = 6). (b) Western blot analysis demonstrating the gp91^*phox*^ expression in the glomeruli of Ctrl mice and DN mice. (c) Summarized data of gp91^*phox*^ protein expression (*n* = 8). Ctrl mice: control mice; DN mice: diabetic nephropathy mice. ^*^ *P* < 0.05 compared with Ctrl mice.

**Figure 3:**
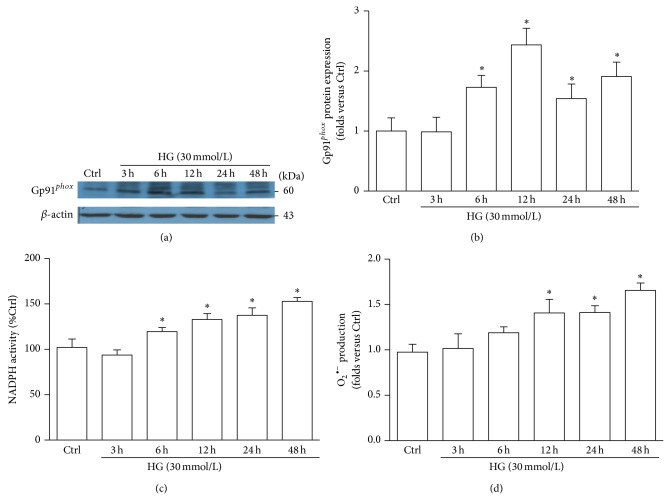
In HG-stimulated podocytes the expression of gp91^*phox*^ is enhanced. (a) Western blotting gel document showing the expression of gp91^*phox*^ in cultured human podocytes without or with HG treatment for indicated time. (b) Summarized data of protein expression of gp91^*phox*^ in podocytes without or with stimulation of HG (*n* = 7). (c) Summarized data showing the effect of HG on NADPH activity in cultured human podocytes (*n* = 5). Data were presented as percent increases in ethidium fluorescence compared to Ctrl. (d) Summarized data showing the fold changes in O_2_^•−^ production, which are normalized to Ctrl (*n* = 7). Ctrl: control; HG: high glucose. ^*^ *P* < 0.05 compared with Ctrl.

ROS is a key regulator for NALP3 inflammasome activation; meanwhile the NADPH oxidase is the main enzyme mediating glomerular ROS generation [7]. Thus, we explored the role of NADPH oxidase in NALP3 inflammasome activation during hyperglycemia. By RT-PCR and western blotting we found that gp91^*phox*^ mRNA and protein were both increased in the glomeruli of DN mice (Figure 2). Consistently, in HG-treated human podocytes the expression of gp91^*phox*^ was elevated in a time-dependent manner (Figures 3(a) and 3(b)). Furthermore, summarized data showed that both NADPH activity and O_2_^•−^ production were also upregulated in HG-stimulated podocytes in a time-dependent manner (Figures 3(c) and 3(d)).

### 3.3. Inhibition of NADPH Oxidase Attenuates HG-Induced NALP3 Inflammasome Activation and Podocyte Injury

**Figure 4:**
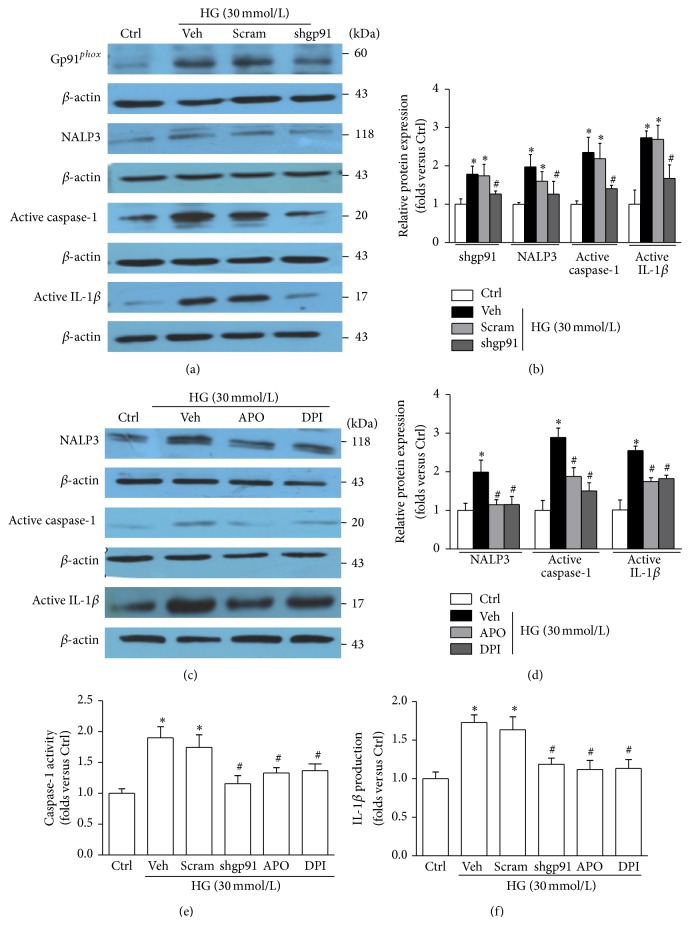
Inhibition of NADPH oxidase attenuates HG-induced NALP3 inflammasome activation and function. (a) Western blot analysis showing the expression of gp91^*phox*^, NALP3, active caspase-1, and active IL-1*β* in HG-stimulated podocytes without or with gp91^*phox*^ shRNA transfection. (b) Summarized data showing the band intensities measured from gp91^*phox*^, NALP3, active caspase-1, and active IL-1*β* (*n* = 6). (c) Immunoblotting presenting the expression of NALP3, active caspase-1, and active IL-1*β* in HG-stimulated podocytes without or with treatment of APO or DPI. (d) Summarized data showing the band intensities of NALP3, active caspase-1, and active IL-1*β* (*n* = 4–8). (e) Caspase-1 activity in podocytes treated with HG in the presence of various genetic and pharmacologic inhibition of NADPH oxidase (*n* = 5). (f) IL-1*β* concentration in the supernatant of podocytes treated with HG in the presence of various genetic and pharmacologic inhibitors of NADPH oxidase (*n* = 6). Ctrl: control; HG: high glucose; Veh: vehicle; Scram: scrambled shRNA; shgp91: gp91^*phox*^ shRNA; APO: apocynin; DPI: diphenyleneiodonium. ^*^ *P* < 0.05 compared with Ctrl. ^#^ *P* < 0.05 compared with HG group treated with vehicle or transfected with scramble shRNA.

**Figure 5:**
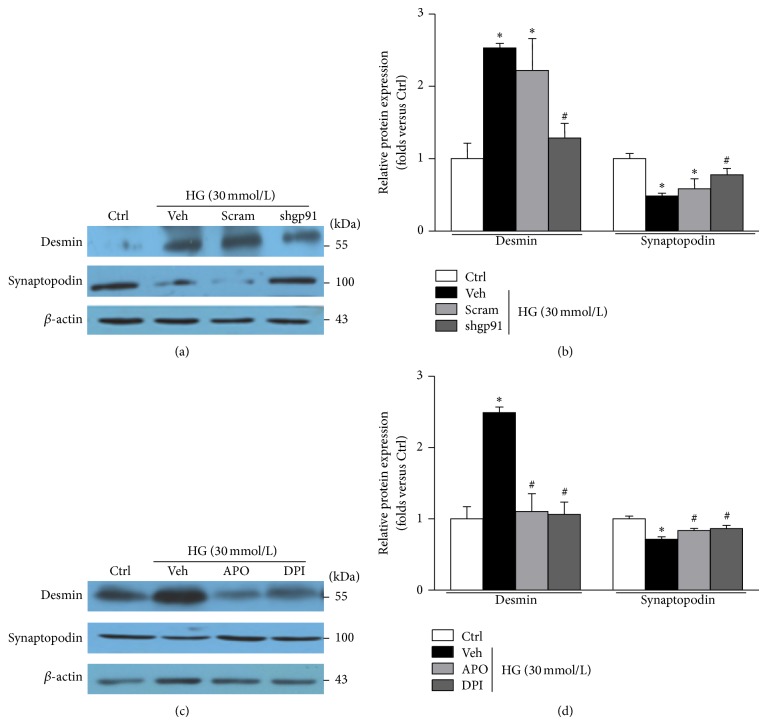
Genetic or pharmacologic inhibition of NADPH oxidase alleviates HG-induced podocyte injury. (a) Western blotting gel documents showing the effect of gp91^*phox*^ gene deletion on desmin and synaptopodin expression in cultured human podocytes with HG treatment. (b) Summarized data of protein expression of desmin and synaptopodin in podocytes (*n* = 4–6). (c) Western blot analysis showing the effect of APO and DPI on desmin and synaptopodin expression in podocytes exposed to HG. (d) Summarized data of the abundance of desmin and synaptopodin protein in HG-exposed podocytes treated with APO or DPI (*n* = 4–8). Ctrl: control; HG: high glucose; Veh: vehicle; Scram: scrambled shRNA; shgp91: gp91^*phox*^ shRNA; APO: apocynin; DPI: diphenyleneiodonium. ^*^ *P* < 0.05 compared with Ctrl; ^#^ *P* < 0.05 compared with HG group treated with vehicle or transfected with scramble shRNA.

As shown in Figures 4(a) and 4(b), silencing gp91^*phox*^ gene expression in podocytes by shRNA alleviated HG-induced accumulation of NALP3 inflammasome components. Accordingly NADPH oxidase inhibitors apocynin (APO) or diphenyleneiodonium (DPI) also blocked HG-induced NALP3 inflammasome activation (Figures 4(c) and 4(d)). As shown in Figures 4(e) and 4(f), the enhanced caspase-1 activity and IL-1*β* production induced by HG were markedly suppressed by gp91^*phox*^ shRNA or by NADPH inhibitors APO/DPI. Thus, our results suggested that NADPH oxidase was significantly implicated in NALP3 inflammasome activation. Furthermore, either genetic deletion or pharmacological inhibition of gp91^*phox*^ halted desmin upregulation and preserved synaptopodin expression in podocytes exposed to HG (Figure 5).

### 3.4. Silencing TXNIP Gene Reduces gp91^*phox*^ Expression

**Figure 6:**
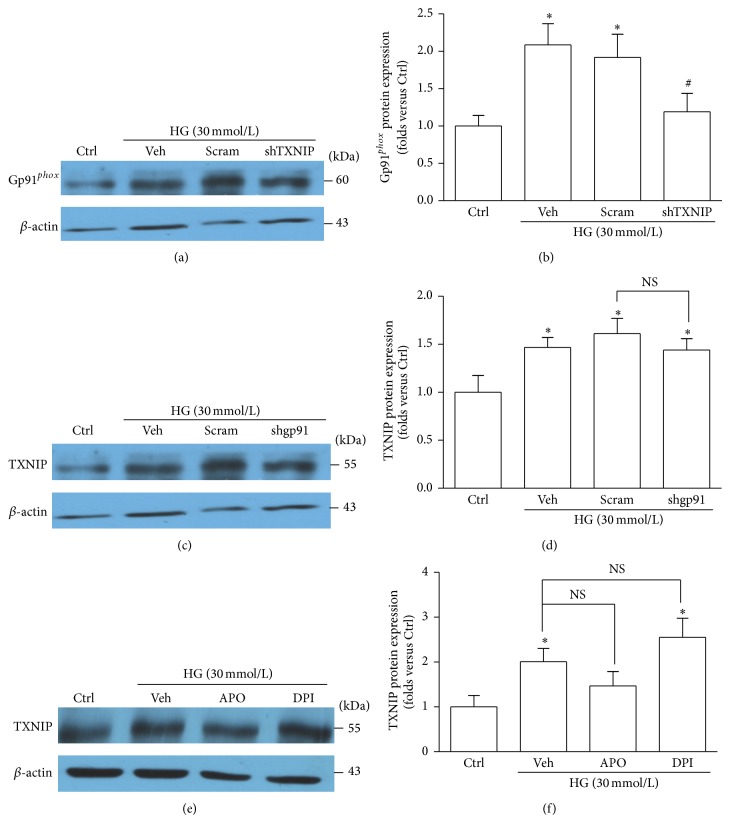
Inhibition of TXNIP abolishes the HG-triggered upregulation of gp91^*phox*^. (a) Western blot analysis showing the expression of gp91^*phox*^ in HG-exposed podocytes without or with TXNIP shRNA transfection. (b) Summarized data showing the band intensities measured from gp91^*phox*^ (*n* = 6). (c) Western blot analysis showing the expression of TXNIP in HG-stimulated podocytes without or with gp91^*phox*^ shRNA transfection. (d) Summarized data showing the band intensities measured from TXNIP (*n* = 6). (e) Protein expression of TXNIP in HG-stimulated podocytes without or with pretreatment of APO or DPI. (f) Summarized data showing the band intensities of TXNIP (*n* = 4–6). Ctrl: control; HG: high glucose; Veh: vehicle; Scram: scrambled shRNA; shTXNIP: TXNIP shRNA; shgp91: gp91^*phox*^ shRNA; APO: apocynin; DPI: diphenyleneiodonium. ^*^ *P* < 0.05 compared with Ctrl; ^#^ *P* < 0.05 compared with HG group treated with vehicle or transfected with scramble shRNA.

In our previous study, we have found that the TXNIP expression was increased in the glomeruli of DN mice and in HG-stimulated podocytes, and silencing TXNIP expression weakened HG-induced NALP3 inflammasome activation and alleviated podocyte injury in cultured human podocytes [27]. Both TXNIP and NADPH oxidase are involved in NALP3 inflammasome activation in HG-stimulated podocytes; however the association between TXNIP and NADPH oxidase is unclear. Here, we proved that the expression of gp91^*phox*^ protein was dampened in podocytes transfected with TXNIP shRNA (Figures 6(a) and 6(b)). Nevertheless, the expression of TXNIP was not affected by gp91^*phox*^ shRNA (Figures 6(c) and 6(d)) or NADPH oxidase inhibitors (Figures 6(e) and 6(f)). Thus, the above observations indicated that TXNIP was required for NADPH oxidase activation in HG-treated podocytes.

### 3.5. Inhibiting TXNIP or NADPH Oxidase Alleviates HG-Induced F-Actin Fibers Reorganization in Podocytes

**Figure 7:**
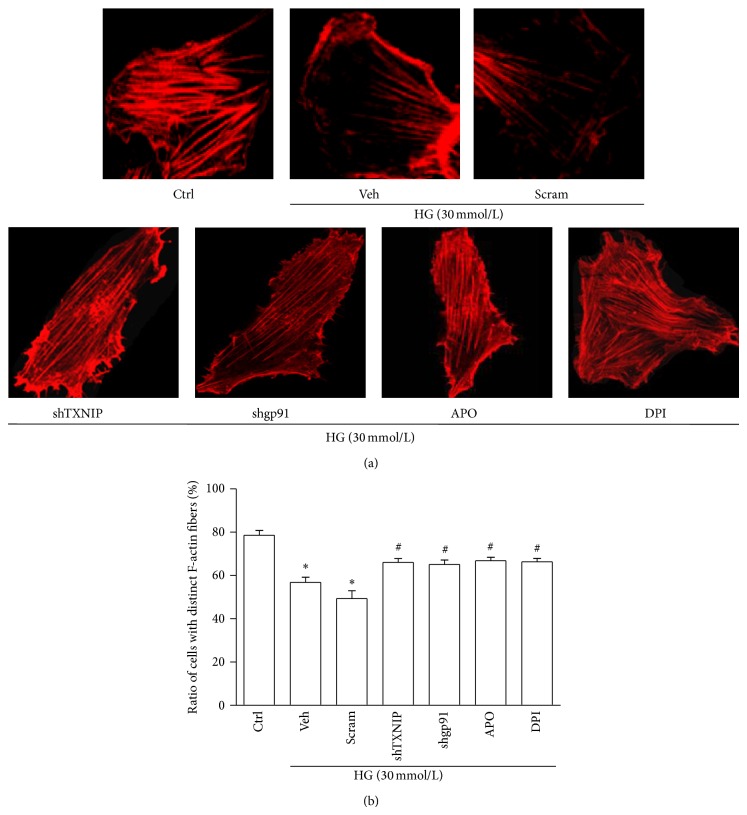
Inhibiting either TXNIP or NADPH oxidase partially preserves the arrangement of F-actin fibers in podocytes exposed to HG. (a) Microscopic images of F-actin by rhodamine-phalloidin staining. (b) Summarized data from counting the cells with distinct, longitudinal F-actin fibers. Scoring was determined from 100 podocytes on each slide (*n* = 5). Ctrl: control; HG: high glucose; Veh: vehicle; Scram: scrambled shRNA; shTXNIP: TXNIP shRNA; shgp91: gp91^*phox*^ shRNA; APO: apocynin; DPI: diphenyleneiodonium. ^*^ *P* < 0.05 compared with Ctrl; ^#^ *P* < 0.05 compared with HG group treated with vehicle or transfected with scramble shRNA.

We finally examined the effect of inhibiting TXNIP or NADPH oxidase on F-actin cytoskeleton structures in podocytes. HG exposure resulted in a loss of the well-defined F-actin fibers which ran along the longitudinal axis of podocytes under normal condition. Interestingly, inhibition of TXNIP or NADPH oxidase partially reversed this alteration in HG-stimulated podocytes (Figure 7).

## 4. Discussion

NALP3 inflammasome activation is involved in the pathological processes of various kidney diseases, including DN, as we and other groups reported [13, 27–32]. But the mechanisms and pathways of NALP3 inflammasome activation in podocytes under hyperglycemia are poorly understood. There are several mechanisms that have been suggested, including ion channel gating, lysosome rupture, and ROS activation [33], among which ROS activation is widely recognized. Under diabetic condition ROS (including NADPH oxidase) has been considered as a key factor accounting for podocyte and glomerular injury [34–37]. And the course of DN was strongly associated with the activity of NADPH oxidase. During hyperhomocysteinemia, hcys could induce podocyte and glomeruli injury via NADPH oxidase-mediated NALP3 inflammasome activation [19], but it is not clear whether it is true under HG milieu. Apart from ROS, we found that TXNIP, a prooxidative and proinflammatory protein, can activate NALP3 inflammasome in HG milieu [27]. Nevertheless, the association between ROS and TXNIP in HG-activated NALP3 inflammasome remains unclear.

Firstly, we hypothesized that NADPH oxidase-induced redox signaling was critical for NALP3 inflammasome activation and the injury of podocytes under hyperglycemia. By immunoblotting we reconfirmed that the components of NALP3 inflammasome were upregulated in the glomeruli of DN mice. Simultaneously the gp91^*phox*^ expression was enhanced in DN mice which was proved by RT-PCR and western blotting. The above observation was further demonstrated in HG-exposed podocytes. In addition, inhibiting NADPH oxidase by genetic or pharmacological strategies alleviated NALP3 inflammasome activation and protected podocytes from HG-induced injury* in vitro.* Thus it is suggested that NADPH oxidase was indispensable for NALP3 inflammasome activation and the damage of podocyte in response to HG. However it still needs to be further evaluated* in vivo*.

As we known, cellular damage caused by redundant ROS is determined by not only the rate of ROS generation, but also the antioxidant defense system [38, 39], which can directly remove ROS or indirectly consume substance generating ROS. Therefore, antioxidant defense system is important for protecting against ROS-associated cell injury. The thioredoxin system belongs to the antioxidant defense system and consists of cytoplasmic thioredoxin (TRX), nicotinamide adenine dinucleotide phosphate-oxidase (NADPH), and homodimeric seleno-protein thioredoxin reductase [40]. TRX is a multifunctional protein that participates in redox-dependent processes, including antioxidant protection from oxidative stress [41]. TXNIP is an endogenous inhibitor of TRX [42] by directly interacting with the catalytic center of reduced TRX and inhibiting its reducing activity [43]. Thus, TXNIP can suppress antioxidant defense mechanism and increase cellular ROS levels [44]. HG could upregulate TXNIP expression and promote oxidative stress [45, 46], which contributes to the pathogenesis of DN in rat and human [47, 48]. Recently Zhou et al. uncovered that TXNIP was essential for hyperglycemia-induced NALP3 activation and caspase-1-dependent IL-1*β* production in the murine pancreatic *β*-cell [16]. In kidney, overexpression of TXNIP in cultured mesangial cells led to increased expressions of collagen IV [49] and related to the high level of oxidative stress in DN [48]. Also our previous study revealed that TXNIP activated NALP3 inflammasome and in turn impaired podocyte structure and function under HG milieu [27]. Since either TXNIP or gp91^*phox*^ can mediate NALP3 inflammasome activation in HG-treated podocyte, next we were interested in elucidating the relationship between TXNIP and gp91^*phox*^ in NALP3 inflammasome activation. We found that the gp91^*phox*^ expression was lessened by TXNIP shRNA in HG-exposed podocytes, whereas the abundance of TXNIP was not affected by NADPH oxidase intervention. Thus we concluded that under hyperglycemia TXNIP accumulation was an upstream event for the following NADPH oxidase (gp91^*phox*^) activation in podocytes.

In summary, our study demonstrated that under hyperglycemia TXNIP-driven NADPH oxidase (gp91^*phox*^) upregulation is accounted for NALP3 inflammasome activation ensuing podocytes injury. This finding provides a new mechanism of podocyte injury in DN which will help us to develop more effective therapies for DN.
