# Erratum to “NADPH Oxidase-Induced NALP3 Inflammasome Activation Is Driven by Thioredoxin-Interacting Protein Which Contributes to Podocyte Injury in Hyperglycemia”

**DOI:** 10.1155/2016/1213892

**Published:** 2016-01-13

**Authors:** Pan Gao, Fang-Fang He, Hui Tang, Chun-Tao Lei, Shan Chen, Xian-Fang Meng, Hua Su, Chun Zhang

**Affiliations:** ^1^Department of Nephrology, Union Hospital, Tongji Medical College, Huazhong University of Science and Technology, Wuhan 430022, China; ^2^Department of Neurobiology, Tongji Medical College, Huazhong University of Science and Technology, Wuhan 430030, China

In Figure 6 in the paper titled “NADPH Oxidase-Induced NALP3 Inflammasome Activation Is Driven by Thioredoxin-Interacting Protein Which Contributes to Podocyte Injury in Hyperglycemia” [1], Figures 6(a) and 6(c) are completely the same pictures. This correction does not have any influence on the results and discussions of the study, which are correctly described in the text. In the published version of Gao et al., 2015, Figure 6(a) should be the representative Western blot graph of the expression of gp91^*phox*^ in HG-exposed podocytes without or with TXNIP shRNA transfection. Below is the correct version of Figure 6.

## Figures and Tables

**Figure 6:**
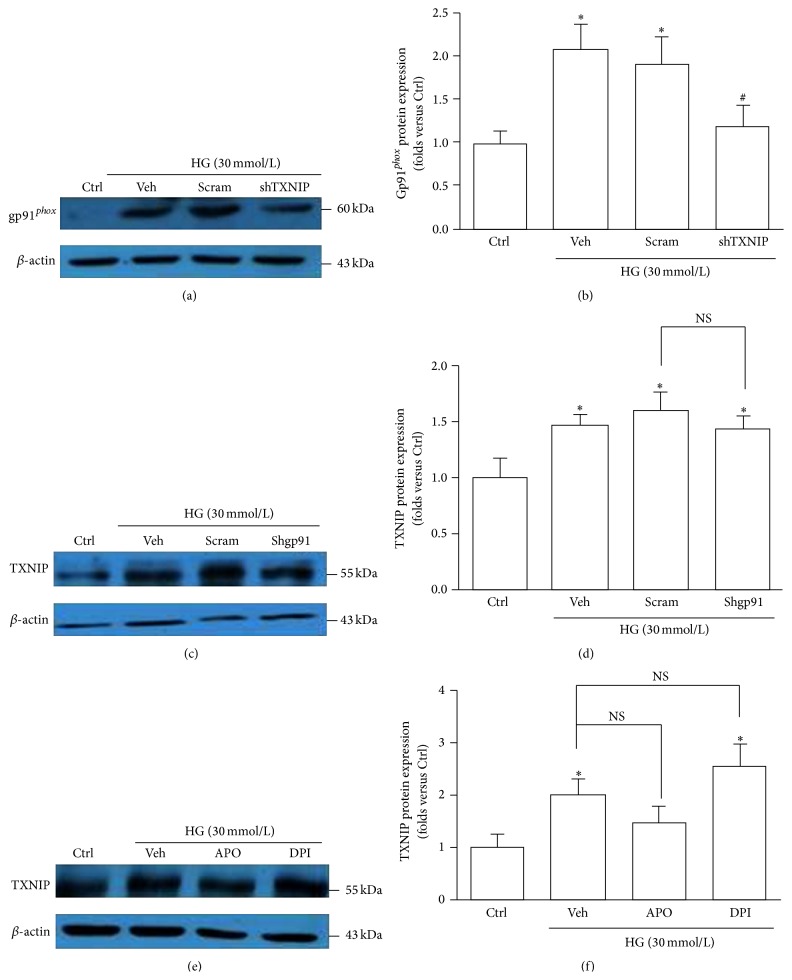
Inhibition of TXNIP abolishes the HG-triggered upregulation of gp91^*phox*^. (a) Western blot analysis showing the expression of gp91^*phox*^ in HG-exposed podocytes without or with TXNIP shRNA transfection. (b) Summarized data showing the band intensities measured from gp91^*phox*^ (*n* = 6). (c) Western blot analysis showing the expression of TXNIP in HG-stimulated podocytes without or with gp91^*phox*^ shRNA transfection. (d) Summarized data showing the band intensities measured from TXNIP (*n* = 6). (e) Protein expression of TXNIP in HG-stimulated podocytes without or with pretreatment of APO or DPI. (f) Summarized data showing the band intensities of TXNIP (*n* = 4–6). Ctrl: control; HG: high glucose; Veh: vehicle; Scram: scrambled shRNA; shTXNIP: TXNIP shRNA; shgp91: gp91^*phox*^ shRNA; APO: apocynin; DPI: diphenyleneiodonium. ^*∗*^ *P* < 0.05 compared with Ctrl; ^#^ *P* < 0.05 compared with HG group treated with vehicle or transfected with scramble shRNA.
